# Experiences of access and use of primary health care by users with systemic arterial hypertension

**DOI:** 10.1590/1980-220X-REEUSP-2024-0109en

**Published:** 2024-10-28

**Authors:** Amanda Maria Vilas Boas Ribeiro, Ana Luiza Queiroz Vilasbôas, Patty Fidelis de Almeida

**Affiliations:** 1 Universidade Federal da Bahia, Instituto de Saúde Coletiva, Salvador, BA, Brazil.; 2 Universidade Federal Fluminense, Instituto de Saúde Coletiva, Niterói, RJ, Brazil.

**Keywords:** Primary Health Care, Hypertension, Access to Health Services, Atención Primaria de Salud, Hipertensión, Accesibilidad a los Servicios de Salud

## Abstract

**Objective:**

To identify and analyze users’ perceptions of access and use of health services and actions to monitor Systemic Arterial Hypertension (SAH) in Primary Health Care (PHC).

**Methods:**

This is a qualitative, descriptive and exploratory study based on 38 semi-structured interviews conducted with users selected from Basic Health Units (BHUs) in a large municipality in the state of Rio de Janeiro. Thematic content analysis was used to identify the empirical categories.

**Results:**

Users faced difficulties in scheduling appointments, accessing laboratory test results and medicines at the BHU. Home visits by Community Health Workers (CHWs) intermediated demands for appointments and tests. There were no health education activities and follow-up by the nursing team was residual. The doctor was the main reference, although links were weakened by turnover.

**Conclusion:**

The study identified the need to expand access to basic medicines and test results at the BHU, the role of nurses in promotional, preventive and clinical actions, the scope of CHWs’ work in health education and interprofessional work.

## RESUMEN

**Objetivo:**

Identificar y analizar las percepciones de los usuarios sobre el acceso y utilización de los servicios y acciones de salud para el seguimiento de la hipertensión arterial sistémica (HAS) en Atención Primaria de Salud (APS).

**Método:**

Se trata de un estudio cualitativo, descriptivo y exploratorio, basado en 38 entrevistas semiestructuradas realizadas a usuarios seleccionados de Unidades Básicas de Salud (UBS) de un gran municipio del estado de Río de Janeiro. Se utilizó el análisis temático de contenido para identificar las categorías empíricas.

**Resultados:**

Los usuarios tuvieron dificultades para programar citas, acceder a los resultados de las pruebas de laboratorio y a los medicamentos en las UBS. Las visitas domiciliarias de los Agentes Comunitarios de Salud (ACS) sirvieron de intermediarios en la demanda por citas y pruebas. No había actividades de educación sanitaria y el seguimiento por parte del equipo de enfermería era residual. El médico era el principal referente, aunque los vínculos se debilitaban por la rotación.

**Conclusión:**

El estudio identificó la necesidad de ampliar el acceso a medicamentos básicos y resultados de pruebas en las UBS, el papel de la enfermería en acciones promocionales, preventivas y clínicas, el alcance del trabajo de los ACS en educación para la salud y el trabajo interprofesional.

## INTRODUCTION

The last two decades have seen an increase in the global burden of disease due to systemic arterial hypertension (SAH)^(1)^. However, population studies in 90 countries show disparities in access to diagnosis, treatment and control of SAH^(2)^, with the estimated percentage of non-adherence to treatment - which ranged from 27% to 40% between 2010 and 2020 - being more prevalent among low- and middle-income countries^(3)^. International experiences reinforce the importance of a health system based on Primary Health Care (PHC) to improve care for SAH with better rates of diagnosis, treatment and control^(4)^. People with SAH who have a regular source of care with a multidisciplinary team in PHC and coordinated care are more likely to achieve lower blood pressure levels due to therapeutic adherence^(5, 6)^. A study carried out in Sweden showed that, in addition to team-based care, people diagnosed with SAH who had a higher proportion of consultations with nurses in PHC were more likely to achieve blood pressure control targets (OR 1.19 – 95% CI 1.07 to 1.32)^(5)^. In the Brazilian context there has been an increase in PHC coverage over the last decades, widening access to health care and advances in the monitoring of chronic conditions, especially medical care coverage and the use of medication by people with SAH^(1)^. However, there is a heterogeneous pattern with regional discrepancies, which affect the way communities and groups access health services^(7)^. In 2019, around 72% of the population who reported having received medical attention in the last 12 months, 66% of this occurred in public services and of these, 45.8% in Basic Health Units (BHU)^(8)^. Around 2% of the population said they had never had their blood pressure (BP) checked, 10% had never sought health services to monitor it and 30.5% said they did not see a doctor regularly to monitor their SAH, revealing numerous challenges for the proper management of chronic conditions related to the low effectiveness of PHC^(8)^.

SAH control also faces constraints related to poor integration between the points of the Health Care Network (HCN), problems related to accessibility and quality of care, inadequate training of the workforce and an insufficient evaluation culture^(9)^, scarcity of material resources, poor adherence to actions, lack of bonds between professionals and users, difficulties in developing educational practices and planning actions in the territory^(10)^.

Considering the strategic position of PHC in the management of chronic conditions, this article aims to identify and analyze users’ perceptions of access and use of health services and actions to monitor SAH in PHC, in a large municipality in the Southeast region of Brazil. In this article, we consider “use” or “access” as the interaction that occurs between the characteristics of health systems, the population/users and service providers, influenced by various factors, including those related to the organization of health services^(11)^, the focus of this analysis.

## METHODS

### Type of Study

This is a case study with a qualitative, descriptive and exploratory approach, based on semi-structured interviews with users with a diagnosis of SAH selected from a BHU in a large municipality who, through their narratives, expressed their experiences of accessing and using health services and actions developed in PHC.

### Location, Population and Selection Criteria

The study was carried out in a municipality in the Southeast region of Brazil, in the state of Rio de Janeiro, with a population of approximately 500,000 inhabitants, 15% of whom live in areas of social vulnerability, all of whom are covered by Family Health Teams (FHT). In February 2022, when the interviews began, there were 84 FHT working in 43 BHU, covering 33.7% of the population^(12)^. From February to July 2022, 38 face-to-face interviews were carried out in ten of the 43 BHU. The criteria used to select the BHU were those with the highest number of FHT (which ranged from three to six) and distribution among the regional health centers. The choice of BHUs with the largest number of teams was justified by the difficulty of finding users who met the criteria for inclusion in the study in the period after the Covid-19 pandemic and the vacancy/change of professionals due to the public selection process that took place at the time, with a period of transition in the PHC workforce - professionals leaving and those approved being hired with unprepared employment contracts. After selecting the BHU, the participants were identified by the interviewers with the help of the FHT according to the inclusion criteria: having a diagnosis of hypertension and being registered with an FHT; a request for a referral by the PHC for a consultation or specialized examination in the last 12 months prior to the interview (verified in the medical records or log books); being 18 years old or older; and not having any physical or psychological impairment that would prevent them from taking part in the interview, according to an assessment by the respective FHT; and distribution among the municipality’s regional health departments.

### Data Collection

After selection, potential participants were contacted and invited to come to the BHU or consulted about the possibility of conducting the interview at home. The interviews were carried out by previously trained researchers and guided by a semi-structured interview script.

The interview script, which is part of a broader study to analyze the care trajectories of users with SAH, was organized into the following blocks: sociodemographic profile, habits and lifestyle, discovery of SAH, the care received in PHC, the experience with specialized care, other paths taken, care during the Covid-19 pandemic and overall evaluation of the care received. For this article, we analyzed experiences of access and use related to the care received in PHC to monitor SAH. The interviews, which lasted an average of 30 minutes, were recorded and transcribed in full for data analysis and processing. The final number of interviews per BHU was determined based on reaching the theoretical saturation point^(13)^ and distribution among the municipality’s health regions.

### Data Analysis and Processing

This study used thematic content analysis^(14)^. Initially, the patterns or themes were identified and described by fully coding each interview, selecting the relevant excerpts grouped into themes and then interpreting the data. In this way, seven empirical categories were identified relating to the monitoring of SAH in PHC. In order to analyze and present the results, the categories identified were compared to the health actions and services that are part of the scope of care for SAH according to Ministry of Health protocols and documents, supplemented by bibliographic sources (Chart 1).

**Chart 1:** Services and actions for monitoring systemic arterial hypertension in primary health care, 2023.

Action/services	Description
Medical, nursing and dental appointments	Medical consultations are essential for diagnosing, setting therapeutic goals and monitoring patients with SAH, allowing them to reassess their blood pressure levels and treatment, encourage self-care practices and refer them to other health professionals or points in the care network, when necessary^(15)^. Nursing consultations are a fundamental component of care for people with SAH^(16)^. It should take place in a systematized way, including the stages of nursing history, diagnosis, planning, implementation and evaluation^(17)^. In order to provide quality oral health care to people with SAH and meet their needs, it is recommended that at least one dental assessment be carried out annually within the PHC. Subsequent dental appointments are determined by the dental surgeon based on oral health risk^(16)^.
Medicines for controlling hypertension	Drug treatment is essential for controlling blood pressure levels, protecting target organs and preventing renal and cardiovascular outcomes. Monotherapy is usually sufficient in the early stages of the disease. In cases where patients do not achieve adequate blood pressure levels, the dose of the drug should be increased or associated with another antihypertensive^(17)^. It is suggested to prescribe drugs that are included in the National List of Essential Medicines (RENAME) and are available in the basic pharmacy or Popular Pharmacy, for greater patient adherence to drug treatment^(16)^.
Laboratory tests for SAH control	The main objectives of laboratory investigations are to screen for lesions in target organs, stratify the patient’s cardiovascular risk and diagnose diseases associated with hypertension. It is recommended that potassium, creatinine, total cholesterol, HDL, LDL calculation, triglycerides, glucose and analysis of physical characteristics, elements and sediments in urine (type 1 urine) be requested annually^(16)^. Professionals should pay attention to the specific nature of each individual, their therapeutic goals, cardiovascular risk and existing complications in order to indicate the periodicity^(16)^.
Blood pressure measurement at the BHU	Controlling blood pressure levels is essential for improving health indicators and preventing complications and hospitalizations resulting from SAH. Blood pressure should be measured at every appointment and, if necessary, self-measurement of blood pressure at home should be recommended, as a measure of adherence, self-care and a source of additional information on treatment^(16)^.
House calls to monitor people with SAH	House calls (HC) should take place routinely, according to the needs of the families, enabling the development of health promotion activities, disease and illness prevention, health surveillance and educational actions^(18)^. In the case of Community Health Workers (CHWs), home visits are an expression of their presence in the territory and a central feature of their work. It stands out for being one of the ways in which services contact users, with the potential to facilitate the population’s access to the health care network, with the aim of caring, informing, registering, strengthening care, the bond and the user’s articulation with the service^(19)^.
PHC reference professional	The reference professional concentrates responsibility for handling individual, family or community cases, expanding the possibilities of building bonds between individuals. Each user must choose or be assigned a reference professional, whose bond is established collectively as well as individually^(16,17,18)^.
Sources of information on SAH and health education practices	Educational and non-drug therapeutic actions with an interdisciplinary approach and self-care are part of the plan for treating adults with SAH and can be developed individually, during consultations or home visits, or collectively in operative groups, waiting rooms, among others^(16)^. They are necessary in order to stimulate lifestyle modification actions such as reducing body weight, stopping smoking, combating stress factors, carrying out regular physical activity, reducing salt and alcohol intake^(15)^. These activities should be carried out by the entire PHC team^(18)^ with the aim of controlling the disease, increasing therapeutic adherence and user autonomy.

Source: Authors, based on Barroso et al.^(15)^, Brasil^(16, 18)^, Cofen^(17)^, Kessler^(19)^.

Acronyms: HAS - Systemic Arterial Hypertension; RENAME - National List of Essential Medicines; CHW - Community Health Workers; LDL - Low Density Lipoprotein; HDL - High Density Lipoprotein; PHC - Primary Health Care

### Ethical Aspects

The study followed all the guidelines and legal prerogatives established by Resolutions 466/2012 and 510/2016 of the National Health Council. It was cleared by the Research Ethics Committee of the Institute of Human Sciences under opinion no. 4.456.756 in 2020, with the consent of the municipality. All participants signed an informed consent form. The participants’ narratives throughout the text have been identified by the letter P and number, according to the order in which the interviews took place.

## RESULTS

### Characterization of the Study Participants

Among the 38 participants, the majority were female (76.3%), self-declared brown or black (71%), aged over 60 (55.3%), without a spouse (63.2%) and with 1 to 2 children (50%). The majority were the main breadwinners (60.5%), with a family income of up to 1 minimum wage (52.6%). Half of the participants (50%) received some kind of social or welfare benefit. Of this total, 36.8% received a municipal social benefit such as a cash transfer (Table 1).

**Table 1:** Characterization of study participants, large municipality, Rio de Janeiro/RJ, Brazil, 2022.

Socioeconomic aspects	N	%
**Age range**		
<49 anos	6	15,7
50–59 anos	11	28,4
60–69 anos	13	34,2
70–79 anos	7	18,4
80 anos ou mais	1	2,6
**Race/Color (self-reported)**		
White	11	28,4
Brown	18	47,3
Black	9	23,6
Indigenous	0	−
Yellow	0	−
**Gender**		
Men	8	21,1
Women	29	76,3
Transvestite	1	2,6
**Schooling**		
Primary incomplete	11	28,4
Primary complete	11	28,4
High School incomplete	5	13,2
High School complete	10	26,3
High Education	1	2,6
**Marital status**		
With partner	13	34,2
Without partner	24	63,2
I prefer not to respond	1	2,6
**Children**		
No children	5	13,2
1 – 2 children	19	50,0
3 or more children	14	36,8
**Main breadwinner**		
Main breadwinner	23	60,5
Other Family member	12	31,6
Shared	2	5,3
Do not say/do not know	1	2,6
**Family income in minimum wages (MW)**		
<1	20	52,6
1–2	10	26,3
Up to 3	5	13,2
Up to 4	2	5,3
Declare no income	1	2,6
**Social benefits**		
No	19	50
Yes Which one?	19	50
*Retirement/Pension*	7	36,8
*Continuous Cash Benefit (BPC)*	1	5,3
*Municipal Social Benefit*	7	36,8
*‘Auxílio Brasil’/Emergency Aid*	4	21,1

Source: Authors, based on the interviews.

The following results are presented in accordance with the health actions and services recommended for monitoring SAH, as shown in Chart 1.

### Medical, Nursing and Dental Appointments at the Phc: There’s Nothing Right for us...

Appointments at the BHU were made possible by face-to-face scheduling. The narratives characterized this process as “difficult” and unpredictable, with long waiting times between the appointment and the actual consultation, ranging from one to three months. Faced with these difficulties, participants sought “inserted” appointments via spontaneous demand, in an attempt to overcome the long waiting time for scheduled actions. In many cases, they experienced long periods of waiting at the BHU until the scheduled appointments were completed. Other participants pointed out that they only went to the BHU for medical care when there was a sign or symptom that justified attending via spontaneous demand:

*It’s difficult to get an appointment there. They don’t make appointments. They book them and we wait there almost all day. You have to wait a long time, sometimes you can’t wait, you just leave.* (P10)

*I get my blood pressure checked when I’m feeling unwell, I wait for everyone to come in, and then I knock on the door and ask the doctor to take a look at me*. (P20)

A significant proportion of those interviewed reported greater restrictions on access during and after the Covid-19 pandemic. Difficulties were reported in organizing care flows in order to guarantee follow-up of other illnesses:

*After this Covid thing, my dear, it became very difficult. I came here several times and was never seen because my doctor was treating Covid and there was never anyone else to see me. So, it was difficult...* (P19)

*Here at the health center because everything stopped, everything was at the mercy of the pandemic. And all the doctors only had to attend to Covid, the ones who did...* (P13)

The narratives indicated possible obstacles to scheduling appointments, the main ones being the unavailability and turnover of professionals, especially doctors. There were frequent reports of repeating the clinical history at each appointment, as they were faced with new professionals with whom they had no previous contact:

*It changes at that post. There’s never a doctor there to see us, you know? He stays there for two or three months, then leaves or is transferred elsewhere. There’s no doctor there.* (P3)

*You have to explain why there’s one doctor there, and when he arrives there’s another one, and then you have to tell him all over again*. (P10)

In many cases, the replacement of professionals was portrayed as a slow process, since the teams remained without a doctor for months, with appointments being suspended. Barriers to access the appointments were associated with the perception of the PHC’s low level of resolution in monitoring SAH:

*Because there’s no doctor at the health center. So, I’m not even going there. [...] We get there [to the PHC] and there’s no doctor. He only sees us when we get there very badly. Other than that, they don’t see us anymore.* (P3). *The service isn’t bad, but I can’t solve my problems*. (P11)

With regard to nursing consultations to monitor hypertension, the narratives indicated a certain lack of precision in terms of recognizing and differentiating between the roles of this professional and those of the technicians. Among the 38 interviewees, 13 mentioned knowing the nurse, without specifying the type of care. The others said they didn’t know the nurse or described functions generally carried out by nursing technicians, such as measuring blood pressure and blood glucose. More often than not, nursing technicians were reported to be more closely involved.

With regard to dental appointments, two thirds of users reported never having been seen by a dentist at the PHC. In some cases, the absence of an oral health team and materials were pointed out as hindering access:

*I saw a chair there, I said it’s a dentist’s chair. It can treat a skull, it can treat a lot of things, but there’s no dentist.* (P15). *[...] there was nothing in the dentist’s room [...] a lot of people in this clinic and not much service*. (P29)

Two participants reported that “simple” dental procedures were carried out at the BHU, such as dental cleaning, restorations and exodontia, while other “more complex” services were carried out at private dental clinics. The quality of oral health procedures was questioned in some cases:

*[...] my area - which here is by area - was without a dentist. Now I’ve come to see if it’s already booked. [...] What they do here is restorations, extractions. As for prostheses, I have to go outside. So I always do it like this: what you do here, I do here. When it’s finished here or when the doctor says: ‘you can go and have your prosthesis done’, then I go there [.in the private clinic].* (P37)

*[...] only the treatment was like this: you did it one day, the next day it fell off, the mixture (laughs).* (P14)

### Medicines For Sah: oh, Everything is Missing... There’s no Dipyrone, There’s no Blood Pressure Medicine...

Access to medicines in PHC was pointed out as one of the main difficulties in treating hypertension. The most frequent combination of access to medicines was via the Popular Pharmacy (PF), complemented by direct purchase, reported by 16 users. The rest accessed exclusively via PF (7), direct purchase (6) or other combinations (BHU, purchase and PF). In any case, PF proved to be the main way of accessing hypertension medication, albeit incompletely:

*I get these medicines... metformin is through the government program... I always go to the popular pharmacy. Because sometimes you go to the pharmacy and they don’t have any. What you’re entitled to in this program is only metformin, losartan and hydrochlorothiazide. I buy the others.* (P16)

*I go to the pharmacy. Some you can get at the pharmacy for free, well. What isn’t, I have to find my own way and buy.* (P12)

Although obtaining medicines through the popular pharmacy was free of charge, users with prescriptions that were “no longer valid” had to go to the PHC to make an appointment to renew their prescription. In the process, they encountered the same difficulties in accessing appointments, so they ended up buying the medicines outright. Narratives pointed to the commitment of the family budget to the purchase of medicines:

*In fact, my prescription expired a long time ago and I couldn’t renew it. I came to renew it, there was no doctor, so I ended up getting used to buying the medication. And some medications the health system doesn’t provide.* (P11). *Sometimes I don’t even have the money (to buy the medication). Since they took away my [Social Benefit], I often don’t even have it. Sometimes, I have to do odd jobs around to be able to*... (P27)

Faced with so many barriers to access or as a consequence of restrictions, there were reports of abandoning treatment when it was impossible to buy the medication with their own resources. On the other hand, one participant felt that the medication provided by the SUS was “weak” and, for this reason, sought out a specialist in the private sector to monitor and prescribe other medication:

*The medication that SUS gives us doesn’t work for me. I think it’s a weak medication, I don’t think it works for me. So I went to an outside endocrinologist and started seeing him.* (P7)

The use of medication followed different patterns, with frequent reports of discontinuous use in the perception of BP control or forgetfulness, even though they were informed of the need for it. The experience of a hypertensive crisis reinforced the need to adhere to drug therapy:

*I stopped suddenly. My pressure was fine, so I didn’t take it at night anymore. I was taking 50 [mg] morning and evening. After coffee I take 50, losartan. Then I stopped taking it, my pressure went up, I got sick, I went to the (hospital with emergency care).* (P25)

*Right, right, after I fell ill, he (the FHT doctor) said: “You’ll never forget it.”* (P22)

### Laboratory Tests to Monitor Hypertension: God Only Knows When the Results Will Come Back...

Most of the interviewees reported having their laboratory tests taken at their own BHU, and the majority felt that the waiting times between requesting and collecting the biological material were not long. However, almost all of them reported that it took a long time to get the results, which were sent to the BHU. The lack of communication from the FHT led to repeated visits to the service, accompanied by anguish and a feeling of frustration at the lack of feedback. Some interviewees mentioned that the results never arrived and that they forgot they had taken the tests:

*The test, you do it today, it takes four, five months for it to be ready, but nobody comes to your house to talk about it. You have to stop by and ask*. (P29). *We arrive, we stand in line. Then you have your blood taken, and then God knows when the results will come back. If you have any problems, you’ll stay. Then, after the results come back, I don’t even know if I have any more.* (P28)

In the perception of some users, when the results were received, they no longer reflected their state of health and had to be repeated. Faced with successive and recurring experiences of delays in receiving the results, a significant number of them turned to private laboratories, despite the financial cost. In these services, they ended up receiving requests for “packages” of tests:

*There are tests we do, it takes two months and they don’t arrive. Then I go to a private laboratory, it’s easy. It’s expensive, but we have to find a way.* (P4)

*Sometimes I have a sister who always helps me. Then, in order to get it faster, she pays for it to be done privately. She looks for the most suitable place, then I go [...] mainly for blood tests, things like that to find out how I’m doing. And bone calcium, vitamin D.* (P14)

### Measuring Blood Pressure at the Bhu: Only When i Feel a Sign, That’s When i’m Coming...

Of the 38 participants, just over half said they monitored their BP at home using a digital device, and 1/3 said they measured it at the BHU. The majority of users who self-measured did not do so regularly, and did so at the slightest sign or symptom. The maintenance of BP checks at the BHU was generally linked to appointments, taking medication or when there was a sign or symptom:

*I have the device to measure it - I bought the device, both of them: diabetes and blood pressure, I have the device to measure it*. (P23)

*Ah, every three days... when I feel that my blood pressure is high, I go and measure it. [...] At home and sometimes I come here [...] a lot of pain in the back of my head. That nauseous headache, a lot of pain in the back of my neck. Then I feel that my blood pressure is high. Then I go and measure it properly and see that it’s high.* (P36)

### House Calls to Monitor Hypertension: They Come Here Because of the Vaccine, and Sometimes They Come to ask for a Test

Around two thirds of users received a home visit from the CHW. There were few reports of visits by other FHT professionals. When they did, it was in serious cases of Covid-19, heart attacks or some other condition of a family member:

*When I was in a coma, the doctors came to my house, you know? A doctor came first, I was in a coma, I came from the hospital with Covid, I came in a coma. Then the doctor came to visit me three times. (...) I have nothing to complain about at the health center, I’ve always been well looked after.* (P3)

*When I had a heart attack, the doctor came, the doctor who used to work here, Doctor (name of doctor), and the nursing technician who used to work with him, who isn’t here today either*. (P13)

In any case, some users understood that according to the “philosophy” of “Family Health”, they should receive a visit from a doctor at least once a year:

*If it’s a family doctor process, for him to be a family doctor, he has to be there infiltrating my family.* (P29)

Among those who received visits from the CHWs, the main actions reported were: communication about scheduling exams or appointments (referral delivery), arrival of exam results, registration or updating, accompaniment by the nursing technician to carry out the Covid-19 vaccination. Although to a lesser extent, there were reports of CHWs being changed due to public selection and the absence of visits in the aftermath of the pandemic:

*We leave that little paper there (reference), they mark it properly and the (CHW) goes to our house to deliver it.* (P31). *From time to time they come to renew the registrations here, they ask a lot of questions. Lately, they haven’t been. After the pandemic, they didn’t come anymore. It’s practically over now here*. (P22)

One user expressed dissatisfaction with the actions carried out during the CHW’s visit, who according to her report “didn’t do anything, just talked, didn’t measure blood pressure, nothing” (P15). Some reports indicated that the CHWs gave advice on the street, when users met them. On these occasions, they were encouraged to go to the BHU:

*I’d meet her [the CHW] on the street: ‘Hey, how are you?’, she knew everyone’s name, ‘no, I’m fine’, ‘look, go on, eh! Anything, go there. [...] No, no [she didn’t go to the house]. She only met them on the street*. (P23)

### Phc Reference Professional: Everything’s new now, Everything’s Changed...

Around a quarter of the participants mentioned that the doctor was the main reference for monitoring SAH, although due to the public selection process at the time, many professionals were no longer part of the FHT. Next, nursing technicians were also mentioned as a reference, especially for carrying out pre-consultation procedures (measuring blood pressure, anthropometry, capillary glycemia) and direct contact with the population during reception:

*I had [reference professional] in the old management (...). Now it’s all new, everything has changed, I’m not intimate with them, I don’t know them [...].* (P21)

*They change. Doctor, just as I was getting used to her, doctor (FHT doctor) left. Then (FHT doctor) worked here, she was very good [...] When I came, the girl had left, they took her off the team. But they sent her somewhere else, I don’t know where*. (P26)

### Sources of Information About Sah and Health Education Practices: the Person has to, at Least, Listen to you...

The main source of information about hypertension and how to control and treat it, mentioned by around half of the participants, was the doctor. Some users also said that they felt comfortable talking about their health problems with other FHT professionals:

*It’s the doctor and the device that I have, that I use. They tell me how much pressure I have to have*. (P4)

In any case, some users reported difficulties in expressing their complaints and doubts due to the lack of a bond with the new professionals, as well as the perception that they were not very receptive to listening.

*The previous doctors were old doctors who knew us. Nowadays, the doctors don’t know the patients here. So it’s very difficult to talk about certain issues*. (P11)

*If you’re going through something, if you go to a place, the person has to at least listen to you. And unfortunately there are people who don’t listen to you, they just pick up the pen, do it like this, that’s it, it’s over. They don’t even look you in the face*. (P33)

Less frequently, friends, family and people close to them were cited as a source for exchanging information and clarifying doubts. The internet was mentioned by three younger participants: “*I Google everything I want to know.Google is my mind”* (P26). In contrast, nine users reported difficulties in using the internet or in relation to the excess of information, which caused more concern. WhatsApp was cited as a tool for communication and transmitting information about SAH by only two participants:

*Ah, I have a lot of friends (laughs). Then they keep sending me messages on WhatsApp. There are times when I’m like, ‘Oh, I can’t take it anymore. All these people think about is illness. For cholesterol, take this, take that. But I don’t take anything*. (P19)

Almost all of the participants reported that they had never taken part in health education activities at the BHU, nor had they been invited to join any group or collective educational practice. Of the 38 participants, only 2 had taken part in any educational activity specifically about SAH, while the other four had taken part in activities on other topics (Pink October Breast Cancer campaign, Smoking):

*Oh, we used to go for walks. We used to go to the garden and walk. It was so good*. *Interviewer: Did you have a hypertension group? Oh, there was a hypertension group. But a long time ago*... (P5)

Most of the interviewees said they didn’t use any other means to manage their hypertension, apart from drug therapy and medical care. Nine mentioned using teas (lemon balm, lemongrass) or other natural products to “calm down” and eight used religions as a source of support. However, even in these cases, there were no reports of medication or medical care being replaced:

*I’m a member of the church, I go to services, I pray, I ask God for my health, for my life, but the medicines I really take are from here*. (P20). *I’ll give you an example: lemongrass is calming. You make a little tea, I take it. But it doesn’t cure heart problems. These things, only the medicine that the doctor gives yo*u. (P27).

## DISCUSSION

Users’ experiences of accessing and using services and actions to monitor SAH indicate a set of barriers, which characterize restricted access to care in PHC, with difficulties in scheduling medical, nursing and dental appointments, an insufficient supply of medication and a lack of health education actions, aggravated by the post-Covid-19 pandemic context, vacancies and changes in professionals. Getting laboratory test results was delayed or did not occur, even when done in a timely manner. The doctor was the main point of reference for care, although the bonds were frayed by turnover.

Similarly to what was observed in this study, there is a higher prevalence of SAH in people with low schooling and adverse socioeconomic conditions^(8)^. In this sense, even more crucial for maintaining health and quality of life is the availability of a comprehensive and resolutive PHC, in which access to curative, preventive and promotional actions can minimize the numerous social vulnerabilities.

Users faced long waiting times between scheduling and attending medical appointments, with a predominance of appointments on spontaneous demand, especially when there was a sign or symptom, indicating that PHC was sought out when the disease worsened, replacing regular appointments^(20)^. The turnover and unavailability of professionals, especially doctors, have been identified as factors that constitute barriers to access, since they limit the availability and quality of services for users with SAH^(21)^. In addition, turnover weakens the construction of a continuum of care, makes it difficult for patients to adhere to therapy, create bonds with the team and ensure continuity of care^(22)^, which was sought to be tackled in the study scenario by making the employment relationships of FHT professionals more precarious.

In this study, users expressed difficulties in recognizing and differentiating between the actions performed by nurses and technicians, indicating weak or non-existent clinical performance by the former. Furthermore, hypertension care was centered on medical consultations, even though the evidence confirms that systematized monitoring by nurses has the potential to increase adherence, reduce hypertensive crises and change body weight^(23)^, which is a challenge that can be addressed through permanent education strategies and valuing these professionals in PHC.

The results showed a restriction in the provision of dental care in PHC. Access to oral health care among people diagnosed with SAH is reduced due to difficulty in accessing public services, high costs of private services and the presence of edentulism^(24)^. These findings reinforce the need to expand and equalize the FHT and oral health teams in order to reduce health inequalities.

Users were faced with the constant unavailability of medicines at the BHU. In this context, similar to other studies^(25)^, they resorted primarily to the Popular Pharmacy, characterizing a change in the pattern of obtaining medicines - from the BHU to the PF, which is an important field for further studies that seek to analyze the monitoring of chronic conditions. Faced with incomplete and interrupted access to medicines through the public system, they resorted to private pharmacies as a last resort, which greatly compromises the budget of the most vulnerable families.

The waiting times to receive the results of laboratory tests was long, even though the time between the request and the collection of biological material was timely. The problem can be tackled through communication strategies between the FHT and users to communicate the results, minimizing frequent trips to the BHU and repeat tests, sometimes in the private network, which have a negative impact on user satisfaction with PHC services^(26)^.

In this study, BP was measured predominantly by users themselves, with or without the support of family members. In the USA, 55% of patients with SAH or related health problems were found to have a home BP monitor, and approximately half of them shared the results of the monitoring with their doctors^(27)^. However, in this study, irregular measurement was observed, both at home and at the BHU, indicating a failure in an important practice for preventing complications and hospitalizations resulting from SAH^(17)^. Most users reported home visits from CHWs, although a significant proportion had never received one. The other FHT professionals did not make any visits. A study using data from the National Health Survey showed an increase in the number of households that never received a visit from CHWs, from 17.7% in 2013 to 23.8% in 2019^(28)^. Visits, when they did take place, were restricted to a narrow scope and involved brokering appointments, exams and services provided at the BHU, to the detriment of health promotion and disease prevention practices. In this sense, given that a significant proportion of users are visited by CHWs, there is a need to encourage the development of collective and community actions during these contacts.

The doctor was reported as the main source of information on SAH and the reference professional, indicating the hegemony of medical knowledge in health practices and the lack of sharing of care with other professionals such as nurses, which could be encouraged by management with a view to improving adherence and monitoring of SAH^(23)^.

There were few reports of health promotion, prevention and education practices for SAH care, as found in other studies^(29)^. The absence of these actions shows the incompleteness of the care pathway for people with SAH, whose access should not be restricted to curative actions and procedures, disregarding the social determinants and potential of community action in a comprehensive PHC.

The study was limited by the fact that it did not analyze institutional documents, medical records or indicators that could have provided elements for data triangulation. The perspective of other actors such as professionals and managers was not part of the scope of the research. No less important was the period in which the study was carried out - after the Covid-19 pandemic and the hiring of professionals via a public selection process. Both factors aggravated the vacancy and change of professionals, one of the main challenges for regular monitoring of SAH, according to the results presented.

## CONCLUSION

Users’ experiences regarding access to and use of health services and actions for monitoring SAH indicate restrictions in the provision of care by PHC. There were difficulties in accessing appointments, unavailability of medication and access to test results in a timely manner, and a lack of practices to promote and prevent SAH. These findings indicate incomplete control of the disease and contribute to the exacerbation of a chronic condition that is prevalent and sensitive to PHC.

Among the aspects that could be intervened upon and improved in the provision of care for SAH, we recommend facilitating access to basic medicines in the BHU, given that drug treatment is essential for adequate control of SAH; interventions in the regulation of diagnostic tests that make up the routine clinical management of the condition studied; recognizing the active role of nurses in caring for users by strengthening promotional, preventive and clinical actions in the nursing sphere; periodic educational home visits by CHWs and adapting their role in health promotion; encouraging interprofessional work within the FHT, with the production of unique therapeutic plans; as well as developing culturally sensitive health education actions in the territory.
