# Supplementary material for: Experiences of access and use of primary health care by users with systemic arterial hypertension
Source: Rev Esc Enferm USP. 2024 Oct 28;58:e20240109. doi: 10.1590/1980-220X-REEUSP-2024-0109en (PMC11534308; doi:10.1590/1980-220X-REEUSP-2024-0109en)
Supplement: Translation

# Translation: Experiências de acesso e uso da Atenção Primária à Saúde por usuários com hipertensão arterial sistêmica

**DOI:** 10.1590/1980-220X-REEUSP-2024-0109pt

**Authors:** Amanda Maria Vilas Boas Ribeiro, Ana Luiza Queiroz Vilasbôas, Patty Fidelis de Almeida

**Affiliations:** 1 Universidade Federal da Bahia, Instituto de Saúde Coletiva, Salvador, BA, Brasil.; 2 Universidade Federal Fluminense, Instituto de Saúde Coletiva, Niterói, RJ, Brasil.

**Keywords:** Atenção Primária à Saúde, Hipertensão Arterial Sistêmica, Acessibilidade aos Serviços de Saúde

## Abstract

**Objetivo:**

Identificar e analisar a percepção dos usuários quanto ao acesso e uso dos serviços e ações de saúde para acompanhamento da Hipertensão Arterial Sistêmica (HAS) na Atenção Primária à Saúde (APS).

**Métodos:**

Estudo qualitativo, descritivo e exploratório, a partir de 38 entrevistas semiestruturadas realizadas com usuários selecionados em Unidades Básicas de Saúde (UBS) em município de grande porte, no estado do Rio de Janeiro. Foi utilizada a análise temática de conteúdo para identificação das categorias empíricas.

**Resultados:**

Os usuários enfrentavam dificuldades para marcação de consultas, acesso aos resultados dos exames laboratoriais e aos medicamentos nas UBS. As visitas domiciliares dos Agentes Comunitários de Saúde (ACS) intermediavam demandas de marcação de consultas e exames. Não ocorriam ações de educação em saúde e o acompanhamento pela equipe de enfermagem era residual. O médico era a principal referência, embora os vínculos estivessem fragilizados pela rotatividade.

**Conclusão:**

O estudo identificou necessidade de ampliar o acesso aos medicamentos básicos e resultados dos exames nas UBS, o papel dos enfermeiros nas ações promocionais, preventivas e clínicas, o escopo de atuação dos ACS na educação em saúde e o trabalho interprofissional.

## INTRODUÇÃO

Nas últimas duas décadas houve aumento da carga global de adoecimento decorrente da Hipertensão Arterial Sistêmica (HAS)^(1)^. Contudo, estudos populacionais em 90 países apontam disparidades no acesso ao diagnóstico, tratamento e controle da HAS^(2)^, sendo o percentual estimado de não adesão ao tratamento – que variou de 27% a 40% entre 2010 e 2020 – mais alto entre países de renda baixa e média^(3)^.

Experiências internacionais reforçam a importância de um sistema de saúde baseado na Atenção Primária à Saúde (APS) para melhoria de atenção à HAS com melhores taxas de diagnóstico, tratamento e controle^(4)^. Pessoas com HAS que possuem uma fonte habitual de cuidados com equipe multidisciplinar na APS e cuidados coordenados, têm maiores chances de alcançar menores níveis pressóricos em virtude da adesão terapêutica^(5, 6)^. Estudo realizado na Suécia mostrou que, além do cuidado em equipe, pessoas com diagnóstico de HAS com maior proporção de consultas com enfermeiros na APS tiveram maior chance de atingir metas de controle pressórico (OR 1,19 – IC 95% 1,07 a 1,32)^(5)^.

No Brasil, ao longo das décadas, houve um incremento da cobertura da APS que ampliou o acesso aos cuidados em saúde e avanços no acompanhamento de condições crônicas, com destaque para cobertura de atenção médica e uso de medicação pelas pessoas com HAS^(1)^. Todavia, observa-se um padrão heterogêneo e com discrepâncias regionais, que afetam o modo como as comunidades e grupos acessam os serviços de saúde^(7)^. No ano de 2019, cerca de 72% da população informou ter recebido atenção médica nos últimos 12 meses; destes 66% receberam atendimento em serviços públicos, sendo 45,8% em Unidades Básicas de Saúde (UBS)^(8)^. Cerca de 2% da população informou nunca haver aferido a pressão arterial (PA), 10% nunca buscou serviços de saúde para seu monitoramento e 30,5% indicaram não ir regularmente ao médico para acompanhamento da HAS, revelando inúmeros desafios para o manejo adequado das condições crônicas relacionados à baixa efetividade da APS^(8)^.

O controle da HAS enfrenta, adicionalmente, constrangimentos relacionados à débil integração entre os pontos da Rede de Atenção à Saúde (RAS), problemas relacionados à acessibilidade e qualidade do cuidado, inadequada formação da força de trabalho, insuficiente cultura avaliativa^(9)^, escassez de recursos materiais, pouca adesão às ações, falta de vínculo entre profissionais e usuários, dificuldades no desenvolvimento de práticas educativas e no planejamento de ações no território^(10)^.

Considerando a posição estratégica da APS no manejo das condições crônicas, este artigo tem como objetivo identificar e analisar a percepção dos usuários quanto ao acesso e uso dos serviços e ações de saúde para acompanhamento da HAS na APS, em um município de grande porte da região Sudeste do Brasil. Neste artigo, consideramos o “uso” ou “acesso realizado” como a interação que ocorre entre características dos sistemas de saúde, população/usuários e prestadores de serviços, influenciada por diversos fatores, entre os quais aqueles relacionados à organização dos serviços de saúde^(11)^, foco desta análise.

## MÉTODO

### Tipo de Estudo

Trata-se de um estudo de caso com abordagem qualitativa, descritivo e exploratório, com base na realização de entrevistas semiestruturadas com usuários com diagnóstico de HAS selecionados em UBS em um município de grande porte que, por meio das narrativas, expressaram suas experiências de acesso e uso dos serviços e ações de saúde desenvolvidas na APS.

### Local, População e Critérios de Seleção

O estudo foi realizado em um município da região Sudeste do Brasil, no estado do Rio de Janeiro, com população aproximada de 500.000 habitantes, sendo 15% de residentes em áreas de vulnerabilidade social, todas com cobertura por Equipes de Saúde da Família (EqSF). Em fevereiro de 2022, início das entrevistas, atuavam 84 EqSF distribuídas em 43 UBS, responsáveis pela cobertura de 33,7% da população^(12)^.

Foram realizadas 38 entrevistas presenciais, de fevereiro a julho de 2022, em dez das 43 UBS. Utilizou-se como critério para seleção das UBS aquelas com maior número de EqSF (que variou de três a seis) e distribuição pelas regionais de saúde. A opção pelas UBS com maior número de equipes se justificou pela dificuldade de encontrar os usuários que atendessem aos critérios de inclusão no estudo no período pós pandemia de Covid-19 e pela vacância/troca de profissionais em função da seleção pública ocorrida à época, com período de transição da força de trabalho na APS – saída de profissionais e contratação dos aprovados com vínculos de trabalho desprecarizados. Após a seleção das UBS, os participantes foram identificados pelos entrevistadores com auxílio das EqSF de acordo com os critérios de inclusão: ter diagnóstico de HAS e estar cadastrado por uma EqSF; solicitação de referência pela APS para consulta ou exame especializado nos últimos 12 meses anteriores à entrevista (verificado nos prontuários ou livros de registro); ter 18 anos ou mais; e não apresentar nenhum comprometimento físico ou psicológico que impedisse a realização da entrevista, segundo avaliação da respectiva EqSF; e distribuição pelas regionais de saúde do município.

### Coleta de Dados

Após a seleção, os potenciais participantes foram contatados e convidados a comparecerem à UBS ou consultados sobre a possibilidade de realização da entrevista em seu domicílio. As entrevistas foram realizadas por pesquisadores previamente capacitados e guiadas por um roteiro de entrevista semiestruturada.

O roteiro de entrevista, que faz parte de um estudo mais amplo para análise das trajetórias assistenciais de usuários com HAS, estava organizado nos seguintes blocos: perfil sociodemográfico, hábitos e estilo de vida, descoberta da HAS, o cuidado recebido na APS, a experiência com a atenção especializada, outros caminhos percorridos, cuidados durante a pandemia Covid-19 e avaliação geral da atenção recebida. Para este artigo foram analisadas experiências de acesso e uso relacionadas aos cuidados recebidos na APS para acompanhamento da HAS. As entrevistas, com média de 30 minutos, foram gravadas, transcritas na íntegra para análise e processamento dos dados. A finalização do número de entrevistas por UBS foi determinada a partir do alcance do ponto de saturação teórica^(13)^ e distribuição pelas regionais de saúde do município.

### Análise e Tratamento Dos Dados

Neste estudo, foi utilizada a análise temática de conteúdo^(14)^. Inicialmente, procedeu-se com a identificação e descrição dos padrões ou temas a partir da codificação completa de cada entrevista, seleção dos excertos relevantes agrupados em temas e, posteriormente, interpretação dos dados.

Desse modo, identificaram-se sete categorias empíricas relativas ao acompanhamento da HAS na APS. Para análise e apresentação dos resultados, as categorias identificadas foram cotejadas às ações e serviços de saúde que fazem parte do escopo de cuidados à HAS segundo protocolos e documentos do Ministério da Saúde, complementado por fontes bibliográficas (Quadro 1).

**Quadro 1:** Serviços e ações para acompanhamento da Hipertensão Arterial Sistêmica na Atenção Primária à Saúde, 2023.

Ação/serviços	Descrição
Consultas médica, de enfermagem e odontológica	A consulta médica é essencial para diagnóstico, estabelecimento de metas terapêuticas e acompanhamento do paciente com HAS, permitindo reavaliar os níveis pressóricos e o tratamento, estimular práticas de autocuidado e encaminhar para outros profissionais de saúde ou pontos da rede de atenção, quando necessário^(15)^. A consulta de enfermagem é componente fundamental do cuidado às pessoas com HAS^(16)^. Deve ocorrer de forma sistematizada, incluindo as etapas de histórico, diagnóstico, planejamento, implementação e avaliação de enfermagem^(17)^. Com o objetivo de ofertar o cuidado em saúde bucal de qualidade às pessoas com HAS e que contemple suas necessidades, é recomentado a realização anual de, pelo menos, uma avaliação odontológica no âmbito da APS. As consultas odontológicas subsequentes são determinadas pelo cirurgião dentista considerando o risco de saúde bucal^(16)^.
Medicamentos para controle da HAS	O tratamento medicamentoso é essencial para controle dos níveis pressóricos, proteção de órgãos-alvo e prevenção de desfechos renais e cardiovasculares. A monoterapia normalmente é suficiente em estágios iniciais da doença. Nos casos em que os pacientes não atingem os níveis pressóricos adequados deve-se aumentar a dose do medicamento ou associá-lo a outro anti-hipertensivo^(17)^. Sugere-se a prescrição de medicamentos que constam na Relação Nacional de Medicamentos Essenciais (RENAME) e são disponibilizados na farmácia básica ou Farmácia Popular, para maior adesão do paciente ao tratamento medicamentoso^(16)^.
Exames laboratoriais para controle da HAS	A investigação laboratorial tem como principais objetivos: rastrear lesões em órgãos-alvo, estratificar o risco cardiovascular do paciente e diagnosticar doenças associadas à HAS. Recomenda-se solicitação anual de dosagem de potássio, creatinina, colesterol total, HDL, cálculo do LDL, triglicerídeos, glicose e análise de caracteres físicos, elementos e sedimentos na urina (Urina tipo 1)^(16)^. Os profissionais devem atentar para a especificidade de cada indivíduo, suas metas terapêuticas, risco cardiovascular e complicações existentes para indicação da periodicidade^(16)^.
Aferição da pressão arterial na UBS	O controle dos níveis pressóricos é imprescindível para melhoria dos indicadores de saúde, prevenção de complicações e internações decorrentes da HAS. A aferição da pressão arterial deve ser realizada em todas as consultas e, se necessário, deve-se orientar a realização da automedida da pressão arterial em domicílio, como medida de adesão, autocuidado e fonte de informação adicional ao tratamento^(16)^.
Visita domiciliar para acompanhamento das pessoas com HAS	As Visitas Domiciliares (VD) devem ocorrer de forma rotineira, de acordo com as necessidades das famílias, possibilitando o desenvolvimento de atividades de promoção da saúde, prevenção de doenças e agravos, vigilância em saúde e ações educativas^(18)^. No caso dos Agentes Comunitários de Saúde (ACS) a visita domiciliar é a expressão de sua presença no território e uma característica central do trabalho deste profissional. Destaca-se por ser uma das formas de contato dos serviços com os usuários, com potencial para facilitar o acesso da população à rede de atenção à saúde, objetivando cuidar, informar, cadastrar, fortalecer o cuidado, o vínculo e a articulação do usuário com o serviço^(19)^.
Profissional de referência na APS	O profissional de referência concentra a responsabilidade na condução dos casos individuais, familiares ou comunitários, ampliando as possibilidades de construção de vínculo entre os sujeitos. Cada usuário deve escolher ou receber a designação de um profissional de referência, cujo vínculo é estabelecido de modo coletivo e em sua dimensão individual^(16,17,18)^.
Fontes de informação sobre a HAS e práticas de educação em saúde	As ações educativas e terapêuticas não medicamentosas com enfoque interdisciplinar e autocuidado fazem parte do plano para tratamento do adulto com HAS e podem ser desenvolvidas em momentos individuais, nas consultas ou visitas domiciliares, ou coletivos em grupos operativos, salas de espera, entre outras^(16)^. São necessárias para estimular ações de modificação do estilo de vida como redução do peso corporal, interrupção do tabagismo, combate a fatores estressores, realização de atividade física regular, redução da ingestão de sal e álcool^(15)^. Estas atividades devem ser realizadas por toda equipe da APS^(18)^ almejando controle da doença, ampliação da adesão terapêutica e autonomia dos usuários.

Fonte: elaboração própria com base em Barroso et al.^(15)^, Brasil^(16, 18)^, Cofen^(17)^, Kessler^(19)^.

Siglas: HAS – Hipertensão Arterial Sistêmica; RENAME – Relação Nacional de Medicamentos Essenciais; ACS – Agentes Comunitários de Saúde; Lipoproteína de baixa densidade – LDL; Lipoproteína de alta densidade – HDL; Atenção Primária à Saúde – APS.

### Aspectos Éticos

O estudo seguiu todas as diretrizes e prerrogativas legais estabelecidas pelas Resoluções 466/2012 e 510/2016, do Conselho Nacional de Saúde. Obteve aprovação do Comitê de Ética em Pesquisa do Instituto de Ciências Humanas sob parecer n° 4.456.756 no ano de 2020, com anuência do município. Todos os participantes assinaram o Termo de Consentimento Livre e Esclarecido. As narrativas dos participantes ao longo do texto foram identificadas pela letra P e número, conforme ordem de realização das entrevistas.

## RESULTADOS

### Caracterização Dos Participantes do Estudo

Entre os 38 participantes, predominaram indivíduos do gênero feminino (76,3%), autodeclaradas pardos ou pretos (71%), com idade acima de 60 anos (55,3%), sem cônjuge (63,2%) e com 1 a 2 filhos (50%). Em sua maioria, eram os principais provedores do lar (60,5%), com renda familiar de até 1 salário-mínimo (52,6%). Metade dos participantes (50%) recebiam algum tipo de benefício social ou previdenciário. Deste total, 36,8% recebiam benefício social municipal do tipo transferência de renda (Tabela 1).

**Tabela 1:** Caracterização dos participantes do estudo, município de grande porte, Rio de Janeiro/RJ, Brasil, 2022.

Aspectos Socioeconômicos	N	%
**Idade**		
<49 anos	6	15,7
50–59 anos	11	28,4
60–69 anos	13	34,2
70–79 anos	7	18,4
80 anos ou mais	1	2,6
**Raça/Cor (autodeclarada)**		
Branca	11	28,4
Parda	18	47,3
Preta	9	23,6
Indígena	0	–
Amarela	0	–
**Gênero**		
Masculino	8	21,1
Feminino	29	76,3
Travesti	1	2,6
**Nível de Escolaridade**		
Fundamental incompleto	11	28,4
Fundamental completo	11	28,4
Médio incompleto	5	13,2
Médio completo	10	26,3
Superior	1	2,6
**Situação Conjugal**		
Com parceiro	13	34,2
Sem parceiro	24	63,2
Prefiro não responder	1	2,6
**Filhos**		
Sem filhos	5	13,2
1 a 2 filhos	19	50,0
3 ou mais filhos	14	36,8
**Principal provedor do lar**		
Principal provedor	23	60,5
Outro membro familiar	12	31,6
Compartilhada	2	5,3
Prefere não falar/não sabe	1	2,6
**Renda Familiar em salários-mínimos (SM)**		
<1	20	52,6
1–2	10	26,3
Até 3	5	13,2
Até 4	2	5,3
Refere não ter renda	1	2,6
**Benefício social**		
Não	19	50
Sim Qual?	19	50
*Aposentadoria/Pensão*	7	36,8
*Benefício de Prestação Continuada (BPC)*	1	5,3
*Benefício Social Municipal*	7	36,8
*Auxílio Brasil/Emergencial*	4	21,1

Fonte: elaboração própria baseada nas entrevistas.

Os resultados a seguir são apresentados em conformidade às ações e serviços de saúde preconizados para o acompanhamento da HAS, dispostos no Quadro 1.

### Consultas Médicas, de Enfermagem e Odontológicas na Aps: Não Tem Nada Certinho Pra Gente...

A realização de consultas nas UBS era viabilizada por meio de agendamento presencial. As narrativas caracterizavam este processo como “difícil” e imprevisível, com longos tempos de espera entre a marcação e a realização da consulta, variando de um a três meses. Frente às dificuldades, os participantes buscavam consultas via demanda espontânea, para “encaixe”, na tentativa de superar o longo tempo de espera para as ações programadas. Em muitos casos, experimentavam extensos períodos de espera na UBS até a finalização dos atendimentos agendados. Outros participantes apontaram a ida à UBS para atendimento médico somente na presença de algum sinal ou sintoma que justificasse atendimento via demanda espontânea:

*Para marcar uma consulta lá, tá difícil. Não marca, não. Elas encaixam e a gente fica quase o dia todo lá esperando. Tem que esperar muito, às vezes, a gente não pode esperar, vem embora. (P10)*

*A pressão eu marco quando tô me sentindo mal, espero atender todo mundo, depois eu bato lá na porta e peço a doutora para me dar uma olhada. (P20)*

Parte importante dos entrevistados sinalizou maiores restrições de acesso durante e no período pós-pandemia de Covid-19. Foram relatadas dificuldades para a organização dos fluxos assistenciais de forma a garantir o acompanhamento dos demais agravos:

*Depois desse negócio de Covid aí, minha filha, ficou muito difícil. Vim aqui várias vezes, nunca era atendida porque minha médica atendia Covid e nunca tinha outra pessoa para atender. Então foi difícil... (P19)*

*Aqui no posto porque parou tudo, ficou tudo à mercê da pandemia. E os médicos todos só tinham que atender Covid, os que atendiam... (P13)*

As narrativas sinalizavam possíveis obstáculos para o agendamento de consultas sendo a indisponibilidade e rotatividade de profissionais, sobretudo médicos, as principais. Foram frequentes relatos de repetição da história clínica a cada consulta, uma vez que se deparavam com novos profissionais, com os quais não possuíam vínculo ou contato prévio:

*Lá naquele posto muda. Nunca para um médico lá para atender a gente, sabe como é? Fica uns dois, três meses, vai embora para outro lugar ou é vai transferido para outro lugar. Fica sem médico ali. (P3)*

*Tem que explicar por que a gente vai é um, quando chega já é outro, aí tem que falar tudo de novo. (P10)*

A substituição de profissionais, em muitos casos, foi retratada como um processo lento, uma vez que as equipes permaneciam meses sem médico, com suspensão dos agendamentos de consultas. As barreiras de acesso às consultas associavam-se à percepção de baixa resolutividade da APS no acompanhamento da HAS:

*Porque no posto de saúde não tem médico. Aí eu nem tô indo lá também. [...] A gente chega lá [na APS], não tem médico. Só atende a gente quando a gente chega lá muito ruim. Fora disso, não tá atendendo mais. (P3)*

*O atendimento não é ruim, mas eu não consigo resolver os meus problemas. (P11).*

Sobre as consultas de enfermagem para acompanhamento da HAS, as narrativas sinalizavam certa imprecisão quanto ao reconhecimento e diferenciação das funções deste profissional e dos técnicos. Entre os 38 entrevistados, 13 mencionaram conhecer o enfermeiro, sem precisar o tipo de atendimento. Os demais reportaram não o conhecer ou descreviam funções, em geral, desempenhadas pelos técnicos de enfermagem como aferição de pressão e de glicemia. Com frequência, maior vínculo foi reportado em relação aos técnicos de enfermagem.

Em relação às consultas odontológicas, 2/3 dos usuários relataram nunca terem sido atendidos por algum dentista na APS. Em alguns casos, a ausência da equipe de saúde bucal e de materiais foram apontados como dificultadores do acesso:

*Eu vi uma cadeira lá, disse que é de dentista. Pode atender crânio, pode atender um monte de coisa, mas não tem dentista. (P15)*

*[...] não tinha nada na sala do dentista [...] muita gente dentro desse posto e pouco atendimento. (P29)*

Duas participantes relataram a realização de procedimentos odontológicos “simples” na UBS, a exemplo de limpeza dentária, restauração, exodontia, enquanto outros serviços “mais complexos” eram realizados em clínicas odontológicas privadas. A qualidade dos procedimentos de saúde bucal foi questionada em alguns casos:

*[...] a minha área – que aqui é por área – estava sem dentista. Aí agora eu vim ver se já está marcando. [...] O que aqui faz, restauração, extração. Já a prótese eu tenho que fazer fora. Então, eu sempre faço assim: o que faz aqui, eu faço aqui. Quando termina aqui ou quando a médica fala: ‘você já pode ir lá fazer a prótese’, aí eu vou lá [.no privado]. (P37)*

*[...] só que o tratamento era assim: você fazia num dia, no outro dia ele caía, a massa (risos). (P14)*

### Medicamentos Para has: ah, Falta Tudo... Não Tem Dipirona, Não Tem Remédio de Pressão...

O acesso aos medicamentos na APS foi apontado como uma das principais dificuldades para tratamento da HAS. A combinação mais frequente para acesso aos medicamentos foi via Farmácia Popular (FP), complementado pela compra direta, relatada por 16 usuários. Os demais acessaram exclusivamente via FP (7), compra direta (6) ou outras combinações (UBS, compra e FP). De toda forma, a FP se apresentou como a principal via de acesso à medicação hipertensiva, ainda que de forma incompleta:

*Esses remédios eu pego... metformina é pelo programa do governo... Na farmácia popular, eu vou sempre na farmácia popular. Porque às vezes vai na farmácia do posto e não tem. O que tem direito nesse programa é só o metformina, losartana e hidroclorotiazida. Os outros eu compro. (P16)*

*Eu vou na farmácia. Uns que pode pegar na farmácia grátis, bem. O que não é, eu tenho que dar meu jeito e comprar. (P12)*

Embora a obtenção dos medicamentos através da FP se realizasse de forma gratuita, usuários com prescrições “fora do prazo de validade” precisavam recorrer à APS para agendamento de consulta médica para renovar a receita. Nesse processo, esbarravam nas mesmas dificuldades de acesso às consultas, assim, acabavam adquirindo os medicamentos por desembolso direto. Narrativas apontaram o comprometimento do orçamento familiar com a compra dos medicamentos:

*Na realidade, a minha receita expirou há um bom tempo e eu não consegui renovar. Vim renovar, não tinha médico, aí por fim acabei acostumando comprar a medicação. E também algumas medicações o sistema de saúde não fornece. (P11)*

*Às vezes, nem tenho (dinheiro para comprar o medicamento). Como tiraram meu [Benefício Social], muitas vezes nem tenho. Às vezes, tenho que fazer uns bicos por aí para poder... (P27)*

Diante de tantas barreiras de acesso ou como consequência de um acesso restrito, houve relato de abandono do tratamento na impossibilidade de aquisição dos medicamentos com recursos próprios. Por outro lado, uma participante considerava que a medicação disponibilizada pelo SUS era “fraca”, e, por esta razão, buscou um especialista da rede privada para acompanhamento e prescrição de outros medicamentos:

*A medicação que o SUS dá para a gente, para mim, não me serve. Eu acho uma medicação fraca, eu acho que não funciona para mim. Então, procurei um endocrinologista fora e comecei a acompanhar com ele. (P7)*

A utilização dos medicamentos obedecia a diferentes padrões, sendo frequentes os relatos de utilização descontínua na percepção de controle da PA ou por esquecimento, mesmo sendo informados sobre sua necessidade. A experiência de crise hipertensiva reforçava a necessidade de adesão à terapia medicamentosa:

*Parei de repente. A pressão estava boa, não tomei mais à noite. Eu estava tomando de manhã e à noite de 50 [mg]. Depois do café tomo de 50, losartana. Aí parei de tomar, a pressão subiu, passei mal, fui para o (hospital com atendimento de emergência). (P25)*

*Direitinho, direitinho. Depois que eu passei mal, ele (médico EqSF) falou: ‘você nunca mais vai esquecer’. (P22).*

### Exames Laboratoriais Para Acompanhamento da has: só Deus Sabe Quando Vai Chegar o Resultado...

A maior parte dos entrevistados referiu realizar coleta para exames laboratoriais na própria UBS, sendo que a maioria considerou que os tempos de espera entre a solicitação e coleta do material biológico não eram longos. Todavia, a quase totalidade referiu muita demora para acesso aos resultados, que eram encaminhados para as UBS. A ausência de comunicação pela EqSF ocasionava repetidas idas ao serviço, acompanhada por angústia e sentimento de frustração diante da ausência de retorno. Alguns entrevistados mencionaram que os resultados nunca chegavam e que se esqueciam de ter realizado os exames:

*O exame, você faz hoje, leva quatro, cinco meses para ficar pronto, mas ninguém também vai na tua casa falar. Você tem que passar aqui para perguntar. (P29)*

*A gente chega, enfrenta fila. Aí tira o sangue, aí depois só Deus sabe quando vai chegar o resultado. Se você tiver algum problema, vai ficar. Aí depois que o resultado sai, nem sei se eu tenho mais. (P28)*

Na percepção de alguns usuários, quando os resultados eram recebidos, não refletiam mais seu quadro de saúde, sendo necessário repeti-los. Frente às sucessivas e recorrentes experiências de demora para recebimento dos resultados, parte expressiva recorria a laboratórios privados, apesar do custo financeiro. Nestes serviços, acabavam por receber solicitação de “pacotes” de exames:

*Tem exame que a gente faz, leva dois meses e não chega. Aí eu vou no particular, é fácil. É caro, mas a gente tem que dar um jeito. (P4)*

*Às vezes, eu tenho uma irmã que sempre me ajuda, né. Aí para sair mais rápido, ela paga para fazer particular. Procura o lugar mais adequado, aí eu faço [...] principalmente exame de sangue, essas coisas assim para saber como é que eu tô. E cálcio dos ossos, a vitamina D. (P14).*

### Aferição da Pressão Arterial na ubs: só Quando dá Sinal Que eu Venho...

Dos 38 participantes, pouco mais da metade referiu realizar monitoramento da PA em domicílio com uso de aparelho digital, 1/3 sinalizou aferição na UBS. A maioria dos usuários com auto aferição não possuía regularidade, e a realizava diante de algum sinal ou sintoma. A manutenção da verificação da PA na UBS, em geral, estava atrelada às consultas, retirada de medicamentos ou diante de algum sinal ou sintoma:

*Eu tenho o aparelho para medir – que eu comprei o aparelho, os dois: tanto da diabetes como da pressão, eu tenho o aparelho para medir. (P23)*

*Ah, de três em três dias... quando eu sinto que a pressão tá alta, eu vou medir. [...] Em casa e, às vezes, venho aqui [...] muita dor na nuca que dá aqui assim. Aquela dor de cabeça enjoada, muita dor na nuca. Aí eu sinto que a pressão tá alta. Aí eu vou, meço direitinho, vejo que tá alta. (P36)*

### Visita Domiciliar Para Acompanhamento da has: Eles Vieram Aqui Por Causa da Vacina, e às Vezes Vem Trazer Pedido de Exame

Cerca de dois terços dos usuários receberam visita domiciliar realizada pelo ACS. Poucos foram os relatos de visitas realizadas pelos demais profissionais da EqSF. Quando aconteciam, foram em casos graves de Covid-19, infarto ou alguma outra condição de algum membro da família:

*Quando eu estava em coma, os médicos vieram na minha casa, sabe como é? Veio um médico primeiro, que eu estava em coma, eu vim do hospital com Covid, eu vim em coma. Aí o médico veio aqui em casa me visitar umas três vezes. (...) Eu não tenho o que reclamar do posto de saúde, eu sempre fui bem atendida. (P3)*

*Quando eu enfartei o médico foi lá, o médico que tinha aqui, doutor (nome do médico), e a técnica de enfermagem que trabalhava junto com ele, que hoje não se encontra aqui também. (P13)*

De toda forma, alguns usuários compreendiam que pela “filosofia” da “Saúde da Família”, deveriam receber uma visita médica pelo menos uma vez ao ano:

*Se é um processo de médico de família, para ele ser de família, ele tem que tá ali infiltrado dentro minha família. (P29)*

Entre os que recebiam visita dos ACS, as principais ações relatadas foram: comunicação sobre a marcação de exames ou consultas (entrega de referência), chegada de resultados de exames, cadastro ou atualização, acompanhamento do técnico de enfermagem para realização da vacinação Covid-19. Embora em menor escala, houve relato de mudanças de ACS pela seleção pública e ausência de visitas no pós pandemia:

*A gente deixa o papelzinho ali (referência), eles marcam direitinho e a (ACS) vai na casa da gente entregar. (P31)*

*De vez em quando eles vão renovar esses cadastros aqui, eles perguntam muita coisa. Agora, ultimamente, que não tem ido. Depois da pandemia, eles não foram mais. Aqui praticamente parou (P22)*

Uma usuária sinalizou insatisfação com as ações desenvolvidas durante a visita do ACS, que segundo seu relato “não fazia nada, só conversava, não media pressão, nada” (P15). Alguns relatos sinalizavam que as orientações do ACS eram realizadas nas ruas, quando os usuários os encontravam. Nessas ocasiões, eram estimulados a comparecerem à UBS:

*Encontrava com ela [ACS] na rua: ‘E aí, como é que está?’, que ela conhecia o nome de todo mundo, ‘não, tô bem’, ‘olha, vai lá, hein! Qualquer coisinha, vai lá’. [...] Não, não [ela não ia na residência]. Só encontrava na rua. (P23)*

### Profissional de Referência na Aps: Agora tá Tudo Novo, Mudou Tudo...

Cerca de um quarto dos participantes mencionou que o médico era a principal referência para o acompanhamento da HAS, embora em função da seleção pública ocorrida à época, muitos profissionais já não faziam parte das EqSF. Na sequência, técnicos de enfermagem também foram mencionados como referência, sobretudo pela realização dos procedimentos de pré-consulta (aferição de pressão arterial, antropometria, glicemia capilar) e contato direto com a população no acolhimento:

*Tinha [profissional de referência] na gestão antiga (...). Agora tá tudo novo, mudou tudo, eu não tenho intimidade, não conheço [...]. (P21)*

*Muda. Doutora, quando eu estava acostumando com ela, doutora (médica da EqSF) saiu. Aí a (médica da EqSF) trabalhou aqui, muito boa. [...] Quando eu venho, a moça tinha saído, tiraram ela da equipe. Mas mandaram para outro lugar, não sei para onde. (P26)*

### Fontes de Informação Sobre a has e Práticas de Educação em Saúde: a Pessoa tem Que Pelo Menos te Escutar...

A principal fonte de informação sobre HAS com abordagem de seu controle e tratamento, referido por cerca da metade dos participantes, foi o médico. Parte dos usuários também referiu que se sentiam à vontade para conversar sobre os problemas de saúde com outros profissionais das EqSF:

*É o médico e o aparelho que eu tenho, que eu uso. Que eles me dizem quanto eu tenho que ter de pressão. (P4)*

De toda forma, parte dos usuários sinalizou dificuldades para a exposição de suas queixas e dúvidas pela falta de vínculo com os novos profissionais, além da percepção de pouca receptividade à escuta:

*Os médicos anteriores são médicos antigos que conheciam a gente. Hoje em dia, os médicos não conhecem os pacientes daqui. Então fica bem complicado passar certas questões. (P11)*

*Se você tá passando, se você vai num lugar, a pessoa tem que pelo menos te escutar. E infelizmente tem gente que não te escuta, já pega a caneta, faz assim, pronto, acabou. Nem olha no teu rosto. (P33)*

Com menor frequência, amigos, familiares e pessoas próximas foram citados como fonte para a troca de informações e esclarecimento de dúvidas. A *internet* foi relatada por três participantes mais jovens: “*eu pesquiso no Google tudo que eu quero saber. O Google é a minha mente” (P26).* Em divergência, nove usuários referiram dificuldades para utilização da *internet* ou em relação ao excesso de informação, que trazia mais preocupação. O *WhatsApp* foi citado como instrumento de comunicação e transmissão de informações sobre a HAS por apenas dois participantes:

*Ah, eu tenho um monte de amigos (risos). Aí fica mandando nesse zap. Tem hora que eu fico: ‘ah, não aguento mais. Só pensa em doença, essa gente’. Para colesterol toma isso, toma aquilo. Mas eu não tomo nada. (P19)*

A quase totalidade dos participantes relatou nunca ter participado de atividades de educação em saúde na UBS, nem terem sido convidados a integrar algum grupo ou prática educativa coletiva. Dos 38 participantes, apenas dois participaram de alguma atividade de educação, especificamente sobre a HAS, outras quatro, de ações sobre outras temáticas (Outubro Rosa, tabagismo):

*Ah, a gente passeava. Ia no horto, caminhava. Era tão bom. Entrevistador: Tinha grupo de hipertenso? Ah, tinha grupo de hipertenso. Mas há muito tempo atrás... (P5)*

A maioria dos entrevistados referiu não utilizar outros meios para cuidar da HAS, além da terapia medicamentosa e cuidados médicos. Nove mencionaram utilizar chás (erva-cidreira, capim-limão) ou outros produtos naturais para ficarem mais “calmos” e oito a religião como fonte de apoio. Contudo, mesmo nestes casos, não houve relatos de substituição da medicação e dos cuidados médicos:

*Eu sou da igreja, vou no culto, oro, peço a Deus pela minha saúde, pela minha vida, mas os remédios eu tomo mesmo é daqui. (P20)*

*Vou te dar um exemplo: capim-limão é calmante. Você fazer um chazinho, eu tomo. Mas não cura para coisa de coração. Essas coisas, só o remédio que o médico passa mesmo. (P27)*

## DISCUSSÃO

As experiências de acesso e uso dos serviços e ações para acompanhamento da HAS, na experiência dos usuários, sinalizam um conjunto de barreiras, que caracteriza um acesso restrito aos cuidados na APS com dificuldades para o agendamento de consultas médicas, de enfermagem e odontológicas, insuficiente oferta de medicamentos e ausência de ações de educação em saúde, agravados pelo contexto pós pandemia de Covid-19, vacância e troca de profissionais. O recebimento dos resultados dos exames laboratoriais era demorado ou não ocorria, mesmo quando realizado em tempo oportuno. O médico era a principal referência de cuidado, embora os vínculos tenham se esgarçado pela rotatividade.

De modo semelhante ao observado nesse estudo, há maior prevalência de HAS em pessoas com baixa escolaridade e condições socioeconômicas adversas^(8)^. Nesse sentido, ainda mais crucial para manutenção da saúde e qualidade de vida é a disponibilidade de uma APS abrangente e resolutiva, na qual o acesso às ações curativas, preventivas e promocionais possam minimizar as inúmeras vulnerabilidades sociais.

Os usuários enfrentavam longos tempos de espera entre a marcação e a realização das consultas médicas, com predomínio de atendimentos por demanda espontânea, principalmente, diante de algum sinal ou sintoma, indicando a procura pela APS em momentos de agudização da doença em substituição às consultas regulares^(20)^. A rotatividade e indisponibilidade de profissionais, sobretudo médicos, foram sinalizadas como fatores constituintes das barreiras de acesso, uma vez que limitam a disponibilidade e qualidade de serviços aos usuários com HAS^(21)^. Adicionalmente, a rotatividade fragiliza a construção de um cuidado contínuo, dificulta a adesão do paciente à terapêutica, a criação de vínculo com a equipe e a continuidade do cuidado^(22)^, o que se buscava enfrentar, no cenário do estudo, com a desprecarização dos vínculos de trabalho dos profissionais das EqSF.

Neste estudo, os usuários expressaram dificuldades no reconhecimento e diferenciação entre as ações desempenhadas pelo enfermeiro e pelos técnicos, indicando fraca ou inexistente atuação clínica dos primeiros. Outrossim, o cuidado à HAS estava centrado nas consultas médicas, ainda que as evidências ratifiquem que o acompanhamento sistematizado pela enfermagem tem potencial para ampliação da adesão, redução de crises hipertensivas e modificações no peso corporal^(23)^, sendo este um desafio passível de intervenção por meio da estratégias de educação permanente e valorização destes profissionais na APS.

Os resultados apontaram restrição na oferta de cuidados odontológicos na APS. O acesso à saúde bucal entre pessoas diagnosticadas com HAS é reduzido em virtude da dificuldade de acesso aos serviços públicos, altos custos dos serviços privados e presença de edentulismo^(24)^. Tais achados reforçam a necessidade de ampliação e equiparação das EqSF e equipes de saúde bucal para diminuição das iniquidades em saúde.

Os usuários deparavam-se com a indisponibilidade constante de medicamentos nas UBS. Neste contexto, semelhante a outros estudos^(25)^, recorriam prioritariamente à Farmácia Popular, caracterizando uma modificação no padrão de obtenção de medicamentos - das UBS para as FP, sendo este um campo importante para o aprofundamento de estudos que buscam analisar o acompanhamento de condições crônicas. Diante da incompletude e interrupção do acesso aos medicamentos pela via pública, como última alternativa, recorriam às farmácias privadas, o que compromete, sobremaneira, o orçamento das famílias mais vulneráveis.

A espera para recebimento dos resultados dos exames laboratoriais era longa, ainda que o tempo entre solicitação e coleta do material biológico fosse oportuno. O problema pode ser enfrentado a partir de estratégias de comunicação entre a EqSF e os usuários para comunicação dos resultados, minimizando os deslocamentos frequentes à UBS e repetição de exames, por vezes na rede privada, que repercutem negativamente na satisfação dos usuários com os serviços da APS^(26)^.

Neste estudo predominou a aferição da PA pelos próprios usuários, com ou sem apoio de familiares. Nos EUA, verificou-se que 55% dos pacientes com HAS ou problemas de saúde relacionados possuíam monitor doméstico de aferição de PA, e aproximadamente metade destes compartilhavam os resultados da monitorização com seus médicos^(27)^. De toda forma, neste estudo, observou-se aferição irregular, tanto no domicílio quanto das UBS, indicando falha em uma importante prática para prevenção de complicações e internações decorrentes da HAS^(17)^.

A maior parte dos usuários reportou visita domiciliar dos ACS, embora parte importante nunca tenha recebido. Os demais profissionais das EqSF não realizavam visitas. Estudo com dados da Pesquisa Nacional de Saúde apontou aumento de domicílios que nunca receberam visita de ACS de 17,7% em 2013 para 23,8% em 2019^(28)^. As visitas, quando existentes, eram restritas a um escopo reduzido e de intermediação de consultas, exames e serviços prestados nas UBS, em detrimento às práticas de promoção da saúde e prevenção de agravos. Nesse sentido, visto que parte importante dos usuários recebe visita do ACS, argumenta-se a necessidade de incentivar, durante estes contatos, o desenvolvimento de ações coletivas e comunitárias.

O médico foi reportado como principal fonte de informação sobre HAS e profissional de referência, sinalizando hegemonia do saber médico nas práticas de saúde e ausência de compartilhamento do cuidado com outros profissionais como os de enfermagem, o que pode ser incentivado pela gestão com vistas a melhorar a adesão e acompanhamento da HAS^(23)^.

Houve poucos relatos de práticas de promoção, prevenção e educação em saúde para os cuidados da HAS, como encontrado em outros estudos^(29)^. A ausência dessas ações evidencia incompletude da linha de cuidado à pessoa com HAS, cujo acesso não deve se restringir a ações e procedimentos curativos, desconsiderando os determinantes sociais e potencialidades da ação comunitária de uma APS abrangente.

Como limites do estudo, não foram analisados documentos institucionais, prontuários ou indicadores que poderiam aportar elementos para a triangulação de dados. A perspectiva de outros atores como os profissionais e gestores não fizeram parte do escopo da pesquisa. Não menos importante foi o período de realização do estudo – pós pandemia de Covid-19 e de contratação de profissionais via processo seletivo público. Ambos os fatores agravaram a vacância e troca de profissionais, uns dos principais desafios para acompanhamento regular da HAS, conforme os resultados apresentados.

## CONCLUSÃO

As experiências dos usuários em relação ao acesso e uso dos serviços e ações de saúde para o acompanhamento da HAS indicam restrições na oferta do cuidado pela APS. Evidenciaram-se dificuldades de acesso às consultas, indisponibilidade de medicamentos e acesso aos resultados de exames em tempo oportuno, inexistência de práticas de promoção e prevenção da HAS. Esses achados indicam incompletude do controle da doença e colaboram para a agudização de uma condição crônica prevalente e sensível à APS.

Entre os aspectos passíveis de intervenção e melhoria na oferta de cuidados à HAS, recomenda-se facilitar o acesso aos medicamentos básicos nas UBS, dado que o tratamento medicamentoso é essencial para o adequado controle da HAS; intervenções na regulação dos exames diagnósticos que compõem a rotina do manejo clínico da condição estudada; reconhecimento do papel ativo dos enfermeiros no cuidado aos usuários com fortalecimento das ações promocionais, preventivas e clínicas no âmbito da enfermagem; realização periódica de visitas domiciliares com caráter educativo pelos ACS e adequação de seu papel na promoção da saúde; incentivo ao trabalho interprofissional no âmbito das EqSF, com a produção de planos terapêuticos singulares; além do desenvolvimento de ações de educação em saúde culturalmente sensíveis no território.
